# Advancing personalized medicine: key priorities for clinical studies and funding systems based on a Europe–China collaborative Delphi survey

**DOI:** 10.1093/eurpub/ckaf004

**Published:** 2025-01-20

**Authors:** Tommaso Osti, Cosimo Savoia, Sara Farina, Flavia Beccia, Francesco Andrea Causio, Lily Wang, Wenya Wang, Carmen Fotino, Chiara Cadeddu, Walter Ricciardi, Stefania Boccia

**Affiliations:** Section of Hygiene, University Department of Life Sciences and Public Health, Università Cattolica del Sacro Cuore, Rome, Italy; Section of Hygiene, University Department of Life Sciences and Public Health, Università Cattolica del Sacro Cuore, Rome, Italy; Section of Hygiene, University Department of Life Sciences and Public Health, Università Cattolica del Sacro Cuore, Rome, Italy; Section of Hygiene, University Department of Life Sciences and Public Health, Università Cattolica del Sacro Cuore, Rome, Italy; Section of Hygiene, University Department of Life Sciences and Public Health, Università Cattolica del Sacro Cuore, Rome, Italy; Jingyi Alliance Clinical Application, BGI, Beijing, China; Center of Biotherapy, Beijing Tsinghua Changgung Hospital, Beijing, China; Fondazione Telethon, Rome, Italy; Section of Hygiene, University Department of Life Sciences and Public Health, Università Cattolica del Sacro Cuore, Rome, Italy; Section of Hygiene, University Department of Life Sciences and Public Health, Università Cattolica del Sacro Cuore, Rome, Italy; Section of Hygiene, University Department of Life Sciences and Public Health, Università Cattolica del Sacro Cuore, Rome, Italy; Department of Woman and Child Health and Public Health, Fondazione Policlinico Universitario A. Gemelli IRCCS, Rome, Italy

## Abstract

Personalized medicine (PM) has the potential to revolutionize healthcare by delivering treatments tailored to individual patients based on their unique characteristics. However, ensuring its effective implementation presents complex challenges, particularly in terms of long-term sustainability. To address these challenges, the IC2PerMed project fosters collaboration between the European Union and China. This study, building on insights from the project, aims to identify key priorities for advancing PM, focusing on Clinical Studies and Funding Systems, with special attention to optimizing resource management, distribution, and protection to support sustainable development. A two-round Delphi survey was conducted to achieve consensus on common priorities among China and Europe related to Clinical Studies and Funding Systems. Consensus was measured using the Content Validity Index, requiring an agreement level of 80% or higher for item inclusion. The survey identified 20 key priorities in PM research, split evenly between research initiatives and funding mechanisms. Notable priorities include developing technology for deep phenotyping, standardizing methodological approaches, and fostering public–private collaborations. In funding, the emphasis was placed on involving patient voices in research design and establishing synergies among funders to support larger projects. The findings underscore the importance of structured collaboration between Europe and China in advancing personalised medicine. By addressing identified priorities in research and funding, this initiative can significantly enhance the efficacy of PM, ultimately improving healthcare outcomes globally. The study sets a precedent for future international partnerships aimed at fostering innovation in health sciences.

## Introduction

In the past decade, the field of personalized medicine (PM) has witnessed a remarkable evolution, establishing itself as a promising frontier in modern healthcare. This approach integrates diverse health data, including patients’ genetic, environmental, and lifestyle factors, to develop more precise and effective interventions [1]. By tailoring diagnostic and therapeutic strategies to individual characteristics, PM has the potential to revolutionize healthcare, improving outcomes while reducing costs [2].

The successful implementation of PM requires the consideration of multiple elements. Among these, research and clinical studies are fundamental pillars, providing the scientific evidence needed to tailor medical interventions to individual patient needs [3]. By analysing genetic, environmental, and lifestyle factors, these studies enable the development of targeted therapies that improve treatment outcomes and reduce adverse effects. Continuous research also fosters innovation by identifying biomarkers and predictive tools that guide more accurate diagnosis, prognosis, and treatment selection [4]. Equally important is the evaluation of funding systems, which are essential to sustain the growth of PM by ensuring that research efforts transition from the laboratory to clinical practice. Public and private investments drive innovation, support the development of new technologies and therapies, and facilitate large-scale clinical trials. Structured funding mechanisms are also crucial for ensuring equitable access to PM treatments, preventing disparities, and enabling healthcare systems to adopt novel approaches sustainably. In both areas, promoting international dialogue plays a key role by facilitating the exchange of best practices and experiences, while supporting the creation of scientific collaborations and funding opportunities [5].

In this context, the European Commission Horizon 2020 programme has funded the ‘Integrating China in the International Consortium for Personalised Medicine’ (IC2PerMed) project, seeking to enable the development of a shared strategy for PM research, innovation, development, and application between the EU and China [6, 7]. The present work, created as part of this project, seeks to identify, through a Delphi survey of experts, priorities relating to the implementation of research and clinical initiatives to promote PM, as well as investments and funding for translational research.

## Methods

Three online workshops were organized to identify priority areas in PM within the IC2PerMed project. The topics selected for these workshops were based on the key priorities outlined in the *ICPerMed Vision for 2030*, combined with insights from a comprehensive mapping exercise on PM policies and initiatives across Europe and China [8]. Detailed mapping results are available in the project’s public deliverables on the IC2PerMed website, along with related scientific publications [9].

This article focuses on the outcomes of one specific workshop, which explored key questions regarding Research and Clinical studies in PM. A combination of bottom-up and top-down approaches was employed to recruit 47 experts for the workshop—37 from the EU and 10 from China. The selection process, detailed in the project deliverable ‘List of WG members and working procedures’ [10], involved identifying experts through publications, partnerships, and projects (top-down) and an open call for experts via the project’s website and communication activities (bottom-up). Although the differing response rates between countries initially raised concerns, the variation was attributed to cultural factors and other considerations, without significantly affecting the results [11]. Before the workshop, participants received guiding questions derived from the mapping exercise of European and Chinese PM policies [12–15]. Following the preliminary discussions, a two-round Delphi expert survey was conducted to reach consensus on key topics [16]. The survey contained 14 items on Research and Clinical Studies in PM and 11 on Research Funding. To ensure clarity and avoid language barriers, the survey was initially drafted in English and then translated into Chinese by an authorized translator.

Experts with specialized knowledge evaluated the content and relevance of each item using a five-point Likert scale (1 = strongly disagree, 5 = strongly agree). Participants could also suggest additional priorities or provide feedback via open-ended questions. After the first round, the survey was revised based on expert feedback and redistributed for the second round. The Delphi process took place maintaining confidentiality and asking participants to declare any potential conflicts of interest.

Consensus was measured using the Content Validity Index (CVI) [17], which reflects the proportion of experts rating an item 4 or 5 out of the total respondents. CVI scores range from 0 to 1 (or 0%–100%). In line with the scientific literature, an agreement level of 80% or higher was required to include an item, while scores between 70% and 79% indicated a need for revision, and scores below 70% suggested removal [18, 19]. The final set of priorities was established through this process, though no specific ranking was assigned—all priorities were considered equally important.

## Results

**Figure 1.**
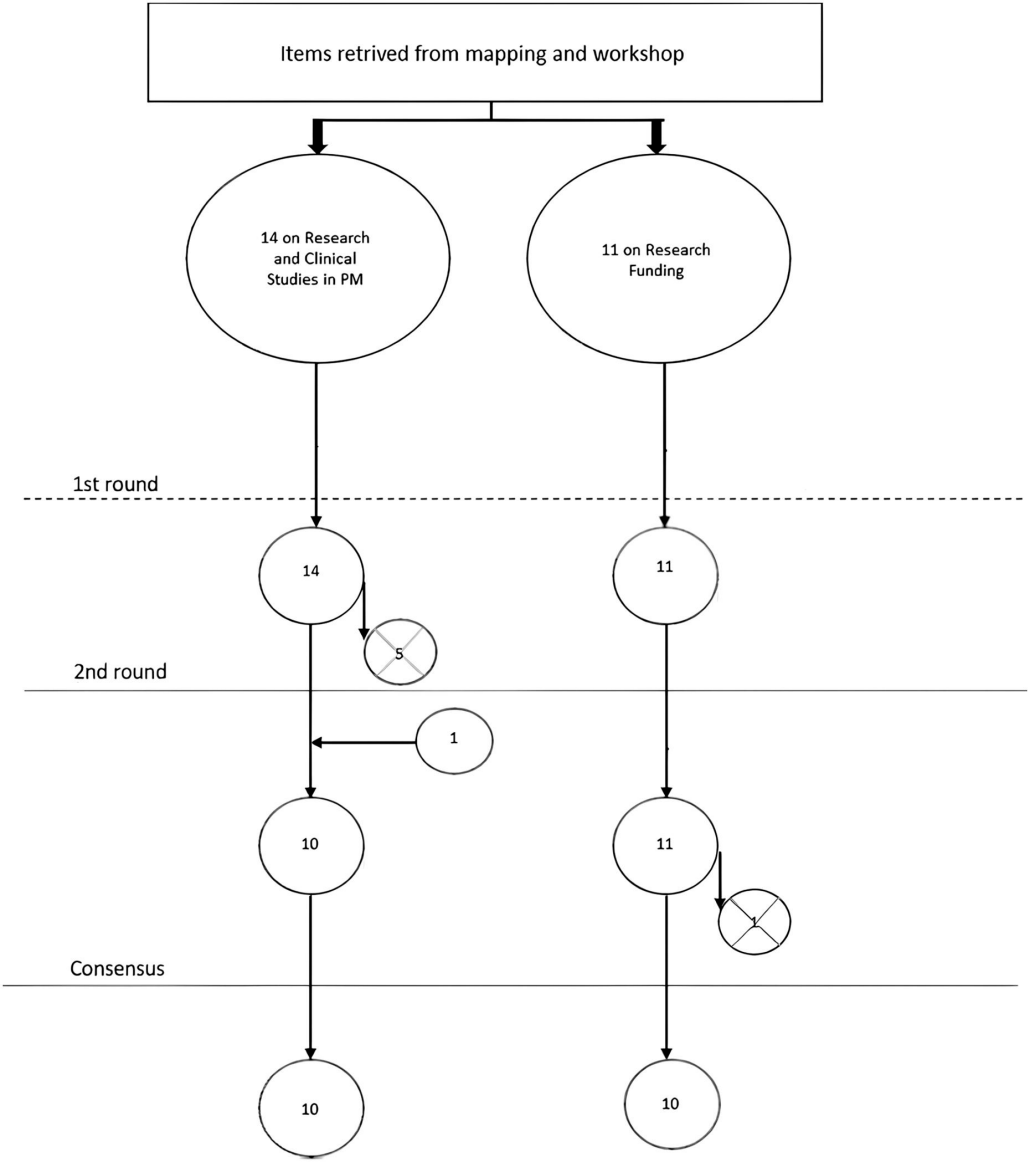
Flowchart of the Delphi survey process. The vertical progression represents the number of priorities before and after each round of the Delphi survey. The circles with an X symbolize the priorities eliminated after each survey round. Horizontal arrows indicate the priorities that were added based on experts’ suggestions of valuable inputs from the WG3 workshops.

A total of 20 priorities have been identified through the Delphi process: 10 for ‘Research and Clinical studies in PM’ and 10 for ‘Research Funding’. The Delphi survey flowchart is detailed in Fig. 1, and the final priorities are detailed in Table 1.

**Table 1.** IC2PerMed priorities on ‘Research and Clinical Studies’ and ‘Funding Systems’

IC2PerMed Priorities on ‘Research and Clinical Studies in Personalised Medicine’
1. Develop and implement technology and standards for deep phenotyping in order to be able to gather patients with similar phenotypes
2. Implementing standardized methodological approaches in PM research to allow patients stratification
3. Promoting exchanges and dialogue among regulatory agencies and research groups to overcome regulatory issues in PM, in particular over the adoption of the most suitable study methods for research in the field of PM and benefit–risk ratio
4. Investing in machine learning algorithm’s research programs to stratify patients in the field of non-genetic/complex diseases
5. Driving applications in epigenomic, metabolomic, and proteomic technologies in addition to genomics for the identification of suitable biomarkers for PM
6. Establishing incentives and public–private collaboration frameworks to facilitate access of academia and industry stakeholders to biological samples and data for research purposes
7. Supporting technical or training workshops/meetings between industry, academia, and biobanks to promote dialogue on EU–China collaboration and understanding of biobanking requirements
8. Creating common projects with the help of funding and governmental agencies to motivate and promote bilateral (EU–China) collaborations and multinational initiatives in PM
9. Using specific disease use cases from fields of rare diseases and cancer to develop international common standards and tools for PM research
10. Fostering controlled access models for clinical trial data sharing to have more standardized PM approaches

IC2PerMed Priorities on ‘Research Funding’

1. Fostering patient voice in all stages of PM research, from the co-design of the research projects to advisory roles and empowering training initiatives to improve awareness of patients and researchers on scientific literacy
2. Implementing exchange of researchers through mobility funding programs to promote collaboration and sharing of knowledge between different countries
3. Funding agencies should tailor investment to patient needs, covering activities on PM implementation and removing barriers to delivery of suitable care to patients
4. Establishing synergies between funders and the research community to implement PM as a community
5. Establishing collaboration among funders to support larger projects, which are bold and spearheading, and enable sharing of risks
6. Investment plays an important role in the whole value chain and is needed from basic science to the implementation of PM in healthcare
7. Invest in the research translation system to overcome the ‘valley of death’ and achieve effective translation and development from academia to industry
8. Creating synergy among funders to align on research topics and to avoid overfocusing on one disease area
9. Harmonizing data sharing among relevant stakeholders to support also non-technology-oriented initiatives
10. Funders should recognize their responsibilities and implications as the first filter on priority setting to ensure appropriate allocation of resources in PM

### Research and clinical studies in PM

Fourteen priorities were identified in the preliminary research. Five priorities were removed after the first Delphi round and one was added after the second round. Ten priorities reached a consensus.

#### Priority 1: Develop and implement technology and standards for deep phenotyping in order to be able to gather patients with similar phenotypes

Validating biomarkers in PM presents challenges due to the unique biological characteristics of each patient. This necessitates the formation of patient cohorts with similar traits to generate robust evidence. Consequently, establishing a global system to identify patients with comparable characteristics could facilitate evidence generation and support the validation of novel biomarkers.

#### Priority 2: Implementing standardized methodological approaches in PM research to allow patients stratification

Patient stratification in PM research is crucial for identifying subgroups of patients who are likely to benefit from specific interventions. Standardized methodological approaches are needed to facilitate accurate patient stratification, improve the reliability and reproducibility of PM research findings, and enable researchers to identify predictive biomarkers, understand disease mechanisms, and develop more targeted interventions.

#### Priority 3: Promoting exchanges and dialogue among regulatory agencies and research groups to overcome regulatory issues in PM, particularly over the adoption of the most suitable study methods for research in the PM and benefit–risk ratio field

Fostering exchanges and dialogue between regulatory agencies and research groups is essential for addressing regulatory challenges in PM. Given that health device and drug regulations are regional rather than universal, understanding the different regulatory frameworks across European Regions and China is crucial. Bilateral exchanges of experiences on communication strategies between regulatory agencies, industry, and academic institutions could create opportunities for mutual growth.

#### Priority 4: Investing in machine learning algorithm’s research programs to stratify patients in the field of non-genetic/complex diseases

While most companies that generate omics data are primarily focused on fields where genomics implementation is more advanced, such as rare diseases and cancer, investing in machine learning research programs for patients’ stratification in non-genetic/complex diseases can help advance our understanding of these conditions and lead to the development of more effective and personalized treatments, as well as improve outcomes for patients.

#### Priority 5: Driving applications in epigenomic, metabolomics, and proteomic technologies in addition to genomics for identifying suitable biomarkers for PM

PM aims to tailor healthcare by adapting treatments to each patient’s unique characteristics. Achieving this requires the identification of reliable biomarkers that can predict individual responses to specific therapies. While genomics has traditionally been the main focus of biomarker discovery, recent advances in epigenomics, metabolomics, and proteomics have opened new pathways for biomarker identification.

#### Priority 6: Establishing incentives and public–private collaboration frameworks to facilitate access of academia and industry stakeholders to biological samples and data for research purposes

Access to biological samples and data is essential for scientific progress, especially in biotechnology. However, these resources are often inaccessible due to regulatory obstacles and limited collaboration between academia and industry. To overcome this, incentives and frameworks for public–private partnerships are needed to facilitate the sharing of samples and data for research. Incentives, such as funding or enhanced data protection regulations, can encourage cooperation, leading to more efficient resource use. Additionally, collaboration frameworks provide structures for resource sharing and expertise exchange, helping to streamline the research process.

#### Priority 7: Supporting technical or training workshops/meetings between industry, academia and biobanks to promote dialogue on EU–China collaboration and understanding of biobanking requirements

Access to biological samples and data is essential for scientific progress, particularly in biotechnology. However, these resources are often difficult to access due to regulatory barriers and limited collaboration between academia and industry. To address this, incentives and frameworks for public–private partnerships are needed to facilitate the sharing of samples and data for research. Incentives such as funding or enhanced data protection regulations can promote cooperation, enabling more efficient resource utilization. Additionally, collaboration frameworks offer structures for resource sharing and expertise exchange, helping to streamline the research process.

#### Priority 8: Creating common projects with the help of funding and governmental agencies to motivate and promote bilateral (EU–China) collaborations and multinational initiatives in PM

Common projects with funding and governmental agencies are required to promote bilateral collaborations and multinational initiatives in PM between the European Union and China. Joint projects can foster collaboration among researchers and institutions by exchanging knowledge, expertise, and resources.

#### Priority 9: Using specific disease use cases from rare diseases and cancer fields to develop international common standards and tools for PM research

Developing international common standards and tools for PM research is critical for ensuring the reproducibility, comparability, and generalizability of findings across disease areas and geographic regions. The use of specific disease use cases from rare diseases and cancer can aid in identifying disease-specific PM biomarkers, pathways, and therapeutic targets, as well as common challenges and opportunities for PM research.

#### Priority 10: Fostering controlled access models for clinical trial data sharing to have more standardized PM approaches

Fostering controlled access models for clinical trial data sharing can aid in developing more standardized approaches to PM research. Controlled access models ensure that data are shared securely and responsibly, while also protecting patient privacy and confidential information.

### Funding systems

Starting from 11 preliminary priorities, 1 priority was eliminated. A consensus was reached on 10 priorities listed in Table 1.

#### Priority 1: Fostering patient voice in all stages of PM research, from the co-design of the research projects to advisory roles and empowering training initiatives to improve awareness of patients and researchers on scientific literacy

Research in PM must involve patients at every stage to satisfy the demands and expectations of patients. Research questions, outcomes, and study design can all be co-designed with patients to help ensure that they are in line with their needs and preferences. Additionally, involving patients in advisory roles and giving them power through training programmes can raise scientific literacy awareness among patients and researchers, fostering a more collaborative research environment.

#### Priority 2: Implementing exchange of researchers through mobility funding programs to promote collaboration and knowledge-sharing between different countries

Implementing researcher exchange programmes through mobility funding is a highly effective strategy for promoting collaboration and knowledge-sharing among different countries. These programmes allow researchers to travel to and work in different countries, which fosters collaboration and allows for knowledge and skill transfer.

#### Priority 3: Funding agencies should tailor investment to patient needs, covering activities on PM implementation and removing barriers to the delivery of suitable care to patients

Funding agencies are vital in advancing PM implementation by providing the necessary resources for research, development, and delivery of PM technologies and services. Tailoring investment to patient needs can promote patient-centred care, improve health outcomes, and increase healthcare delivery efficiency. Removing barriers to the delivery of appropriate care can improve patient access to PM and reduce healthcare disparities.

#### Priority 4: Establishing synergies between funders and the research community to implement PM as community

Creating synergies between funders and the research community is critical to the success of PM as a community effort. PM needs cooperation between researchers, healthcare providers, and funders to achieve its objectives of personalized treatments and improved patient outcomes. The direction of PM research is significantly influenced by funding organizations, and by collaborating closely with the research community, they can make sure that funds are allocated to the most exciting areas of study.

#### Priority 5: Establishing collaboration among funders to support larger projects, which are bold and spearheading, and enable sharing of risks

Collaboration among funders is essential for supporting larger, innovative, and modern projects. By pooling resources and sharing the risks, funders can work towards a common goal, leveraging their individual strengths, expertise, and networks. This approach has a greater impact, as larger projects tend to be more complex and require a broader range of resources than smaller initiatives.

#### Priority 6: Investment plays an important role in the whole value chain and is needed from basic science to the implementation of PM in healthcare

Investment is critical for the entire precision medicine value chain, from basic science to healthcare implementation, and this is still being implemented in China. Private organizations face fundraising challenges, and public–private partnerships should be encouraged.

#### Priority 7: Invest in the research translation system to overcome the ‘valley of death’ and achieve effective translation and development from academia to industry

Investing in the research translation system can help bridge the ‘valley of death’, the gap between academic research and commercial development. This system offers funding and support for research translation across various stages, including proof-of-concept studies, academic work, pre-clinical and clinical trials, and commercialization efforts, such as start-ups and industrial development.

#### Priority 8: Creating synergy among funders to align on research topics and to avoid overfocusing on one disease area

Creating synergy among funders is critical in medical research to avoid overfocusing on one disease area. Funders can share resources, avoid duplication of efforts, and ensure that the most pressing health challenges are addressed by collaborating and aligning on research topics.

#### Priority 9: Harmonizing data sharing among relevant stakeholders to support also non-technology-oriented initiatives

Data sharing between academia and industry is challenging, and combining data from different sources is often complicated by interoperability issues. In public–private partnerships, the pharmaceutical industry is sometimes willing to share placebo arms from clinical trials with academic researchers, providing highly valuable data for analysis. China has been working on data-sharing legislation and has policies promoting the sharing of research data within academia, but access remains limited.

#### Priority 10: Funders should recognize their responsibilities and implications as the first filter on priority setting to ensure the appropriate allocation of resources in PM

Funders are crucial in establishing priorities for allocating resources in PM. They are frequently the first filter through which project proposals are evaluated, and their decisions on which projects to fund can have significant consequences for a project's success or failure. Funders must accept responsibility for prioritizing projects based on potential impact, feasibility, and alignment with organizational goals.

## Discussion

Our study highlights several critical challenges related to the development of PM in clinical studies and funding mechanisms, focusing on both European and Chinese contexts. Key areas include multi-omics applications and biomarker discovery, the role of research infrastructure, the development of advanced therapies, and primary funding sources and priorities for PM research.

The refinement of multi-omics tools and biomarker discovery is fundamental to PM research [20]. These technologies must strike a balance between capturing individual-specific traits and ensuring that these traits are shared among population subgroups, enabling public health interventions [21]. Phenotyping plays a crucial role in collecting observable traits; however, traditional methods relying on subjective researcher criteria are inadequate for PM [22]. Deep phenotyping, defined as gathering detailed data on disease manifestations and integrating it with other information through advanced algorithms [23], offers a more precise grouping of patients based on traits such as genetic and epigenetic profiles. This facilitates the validation of biomarkers and multi-omics tools. However, criteria for patient stratification remain fragmented between China and Europe, limiting the efficient use of data. Harmonizing these criteria would allow global data integration, as highlighted by the Horizon 2020-funded PERMIT project, which stressed the need for stakeholder discussions to establish best practices across diseases [24].

The involvement of regulatory agencies in PM research is essential, as PM increasingly moves beyond genomics to incorporate metabolomics, proteomics, and epigenomics, among other fields [25]. These complementary approaches enhance the understanding of biological traits and health prediction, promoting the development of advanced biomarkers and therapies [26]. The integration of diverse omics data generates vast datasets, necessitating the use of computational tools, including machine learning. Machine learning algorithms can identify patterns in high-dimensional data, supporting the creation of predictive models for PM [27].

Research infrastructure plays a pivotal role in supporting PM research in both Europe and China. Strengthening public–private partnerships allows academia and industry to utilize biological data effectively, addressing challenges related to low-prevalence diseases, biomarkers, and high clinical trial costs. Fragmented international regulations complicate research initiation for non-governmental stakeholders, underscoring the need for economic incentives and legal protections to engage all PM actors. Initiatives like the Cancer Moonshot Lund Center, which collaborates with over ten countries through the US Cancer Moonshot Initiative, exemplify the value of academic, industrial, and governmental cooperation in advancing cancer research through shared biological samples and data [28]. Biobanks are critical to PM research, serving as repositories of essential data. These repositories must be integrated into protected information streams and engaged in workshops to enhance best practices in sample and data management, ensuring research quality and reliability. Collaboration between biobanks, academia, and industry advances diagnostics and therapeutic development, benefiting patients worldwide. BBMRI underscores the importance of providing access to samples from the 700 biobanks under its management, supported by robust IT teams to facilitate this access [29]. Strengthening technical workshops among key stakeholders promotes biobanking research and fosters global health. Joint PM projects between Europe and China, with government and funding agency support, can address challenges related to data sharing, standardization, and ethics, ultimately accelerating PM advancements and improving healthcare outcomes [30]. Developing advanced therapies in PM requires targeted strategies, with rare diseases and cancer serving as essential use cases [31, 32]. These examples can expedite the development of standardized tools, data-sharing frameworks, and harmonized ethical regulations. They also offer insights for advancing PM research and enhancing personalized care.

In terms of funding priorities, early involvement of patients in PM research is crucial. Studies show that patient engagement improves satisfaction, outcomes, and research efficiency [33, 34]. Involving patients, even inexperienced ones, in early research phases–such as protocol development—optimizes collaboration. Training programs like EUPATI, which educates patient advocates to engage with researchers, empower patients to make informed health decisions [35].

International mobility programs for researchers also play a vital role in fostering knowledge exchange, collaboration, and innovative solutions to global challenges. The European Commission found that such programs correlate with higher publication rates, increased productivity, and better career prospects [36]. Funding priorities in PM include infrastructure development, such as laboratories, omics technologies, and computational resources for secure data use, as well as personnel training through specialized courses [37].

Collaboration between funders and researchers accelerates the clinical application of PM research, benefiting patients directly. Public health research provides funding agencies with evidence-based insights into specific challenges, including disease prevalence, resource allocation, and health disparities within healthcare settings, facilitating the development of personalized diagnostic tools and therapy while strategically driving impactful innovation. This collaboration also supports translational research, bridging the gap between academic discoveries and practical solutions. Investments in PM come with inherent risks, which can be mitigated through large-scale collaborative projects. Sharing resources, best practices, and expertise ensures sustainable development, as exemplified by the European Partnership on Personalised Medicine [38]. Aligning funding efforts prevents gaps and overemphasis in specific areas of PM research. Public–private partnerships and social financing mechanisms are also essential. China’s ‘Healthy China 2030’ initiative promotes collaboration with social resources to complement public funding [39]. Disease associations and foundations can contribute to funding rare disease research, benefiting both the EU and China. However, public–private collaboration introduces data-sharing challenges. Harmonizing methods across stakeholders and addressing ethical, legal, and cultural barriers is crucial. Ongoing international initiatives aim to develop innovative data-sharing models, e.g. the European Health Data Space, which seeks to create a secure platform for exchanging health data across EU borders to support PM by leveraging real-world data to generate real-word evidence [40]. Funders must also ensure that resources are allocated to projects with high success potential, considering the long-term impact and closely monitoring project outcomes.

The IC2PerMed project emphasizes the need to support new studies in PM to discover innovative biomarkers and therapies. Advancing PM requires ongoing research and a structured funding system to meet evolving diagnostic and therapeutic goals. Collaboration between Europe and China offers valuable opportunities by enabling knowledge exchange and joint funding, accelerating PM advancements. This partnership not only drives research progress but also provides a model for future international cooperation, fostering innovation and improving global healthcare outcomes.

## Data Availability

All the documents mentioned in the manuscript and the workshops’ results are available for consultation at https://www.ic2permed.eu/zh/publications-public-deliverables-2/.
